# Real-World Effectiveness and Safety of Oral Alitretinoin in Chronic Hand Eczema with Exploratory Off-Label Indications: A 10-Year Single-Center Korean Study

**DOI:** 10.3390/medicina62091625

**Published:** 2026-08-24

**Authors:** Jeongsoo Lee, Joonsoo Park

**Affiliations:** Department of Dermatology, School of Medicine, Daegu Catholic University, 33 Duryugongwon-ro 17-gil, Nam-gu, Daegu 42472, Republic of Korea; roun23@cu.ac.kr

**Keywords:** alitretinoin, chronic hand eczema, real-world data, off-label use, palmoplantar pustulosis, mycosis fungoides, dyslipidemia, treatment outcome

## Abstract

*Background and Objectives*: Oral alitretinoin (9-cis-retinoic acid), a dual retinoic acid receptor/retinoid X receptor agonist, is approved for severe chronic hand eczema (CHE) refractory to potent topical corticosteroids but is also prescribed off-label in routine practice. Long-term real-world data on both uses are limited. We evaluated 10-year prescribing patterns, effectiveness, tolerability, and lipid changes at one Korean academic center. *Materials and Methods*: Patients prescribed oral alitretinoin between January 2016 and December 2025 were identified from the hospital prescribing database; those with an evaluable course of ≥1 month formed the effective-treatment cohort and underwent chart review. Treatment success was defined as final chart-derived Investigator Global Assessment (IGA) 0/1. Success by initial dose was compared using Fisher exact test; paired lipid changes using paired *t*-test and Wilcoxon signed-rank test. *Results*: Of 161 patients, 38 (23.6%) received a single prescription; 99 formed the effective-treatment cohort (50 women, 49 men; mean age 50.7 ± 16.0 years). CHE was the leading indication (70/99, 70.7%), followed by palmoplantar pustulosis (PPP, *n* = 14), mycosis fungoides (MF, *n* = 6), parapsoriasis, porokeratosis, palmoplantar keratoderma, and Darier disease. Overall success was 66.7% (66/99) over a mean treatment duration of 12.4 months, highest in CHE (77.1%) and more variable off-label (PPP 42.9%; MF 33.3%). Initiation at 30 mg was associated with higher success than 10 mg (71.1% vs. 43.8%; *p* = 0.044), but this association did not persist after restriction to patients maintained on their initial dose (62.2% vs. 50.0%; *p* = 0.498) or after Firth penalized logistic regression adjusting for age, sex, and indication (adjusted odds ratio 1.84; 95% CI 0.56–5.90; *p* = 0.309). Adverse events occurred in 21 patients (21.2%), most commonly dyslipidemia (*n* = 12) and headache (*n* = 7); mean triglyceride rose 42.1 mg/dL and total cholesterol 14.0 mg/dL (both *p* < 0.001), and new elevation above conventional thresholds occurred in 25.8% (triglyceride) and 27.5% (total cholesterol) of patients with normal baseline values. Thirty patients (30.3%) discontinued, most often for insufficient effectiveness. *Conclusions*: Alitretinoin showed favorable effectiveness in CHE and variable, exploratory responses off-label. The apparent advantage of 30 mg initiation was not independent of indication and dose adjustment. Lipid elevation was common, supporting routine surveillance. Off-label findings, particularly in PPP and MF, should be interpreted cautiously given small subgroups and non-standardized retrospective assessment.

## 1. Introduction

Alitretinoin (9-cis-retinoic acid) is an endogenous retinoid that binds both retinoic acid receptors (RARs) and retinoid X receptors (RXRs). This dual receptor activity provides a biologic rationale for effects on epidermal differentiation, keratinization, inflammation, and lymphocyte-related pathways [1,2]. Pharmacokinetic studies have also shown rapid clearance compared with longer-retained systemic retinoids, a feature that is clinically relevant when selecting retinoid therapy [1].

Oral alitretinoin is approved for severe chronic hand eczema (CHE) that is refractory to potent topical corticosteroids. Approval was supported by randomized, double-blind, placebo-controlled trials in which alitretinoin produced dose-dependent clearing of refractory disease, with higher daily doses achieving greater response rates than lower doses [3,4]. Large real-world studies and meta-analytic data further support clinically meaningful improvement in CHE, including improvements in physician-rated severity, quality of life, work productivity, and patient-relevant symptoms [5,6,7]. In a Korean retrospective study including hand eczema and palmoplantar pustulosis (PPP), response was observed in both conditions, but hyperkeratotic morphology rather than diagnostic label appeared to be the strongest predictor of response [8].

Beyond CHE, alitretinoin has been explored in a range of difficult-to-treat dermatoses. In PPP, evidence remains controversial: a randomized phase II study did not demonstrate superiority over placebo, whereas subsequent interpretation emphasized the unusually high placebo response and the need for larger studies [9,10]. In cutaneous T-cell lymphoma (CTCL), including mycosis fungoides (MF) and Sézary syndrome, several retrospective series have reported response or stable disease with alitretinoin, often in combination with standard skin-directed or systemic therapies [11,12,13]. Additional reports describe potential benefit in refractory prurigo and ichthyosis, particularly when conventional options are limited [14,15]. Isolated reports have also described responses in keratinizing and hyperkeratotic disorders, including refractory palmoplantar keratoderma [16,17], Darier disease [18], and disseminated superficial actinic porokeratosis [19], although the durability of benefit in Darier disease has subsequently been questioned [20].

Most available evidence is disease-specific, short-term, or derived from selected cohorts. Less is known about how alitretinoin is used over long periods in routine clinical practice across both approved and off-label indications. We therefore analyzed 10 years of institutional prescribing and chart-review data to characterize real-world use, effectiveness, tolerability, discontinuation, and lipid changes associated with oral alitretinoin at a Korean academic dermatology center.

## 2. Materials and Methods

### 2.1. Study Design and Data Source

This single-center retrospective study combined drug-utilization analysis with detailed chart review. All prescriptions of oral alitretinoin (Alitoc; institutional product codes DALITO1 and DALITO3, corresponding to the 10 mg and 30 mg capsule strengths, respectively) issued at Daegu Catholic University Medical Center between 1 January 2016 and 31 December 2025 were extracted from the hospital prescribing system. The database extraction identified 1455 prescription records from 161 unique patients.

### 2.2. Cohort Definition

The full prescribing population included all unique patients who received at least one alitretinoin prescription during the study period. Because a single prescription or very short exposure cannot reliably represent a therapeutic course, the effective-treatment cohort was defined a priori as patients with an evaluable treatment course of at least 1 month. This threshold was selected as the minimum exposure required to define a clinically interpretable treatment course and to allow at least one subsequent clinical assessment; it was not intended to indicate that 1 month is sufficient to reach maximal alitretinoin effectiveness, and final response was assessed at the end of the available course rather than at 1 month. Patients with only a single prescription, with shorter exposure, or without final treatment-related clinical documentation sufficient to assign an outcome were described at the drug-utilization level but excluded from treatment-outcome analysis, because response, non-response, and adverse-event attribution could not be classified reliably from such limited exposure or documentation. Characteristics of the excluded groups are summarized in Supplementary Table S1. The final effective-treatment cohort comprised 99 patients who underwent chart review.

### 2.3. Diagnosis Adjudication and Outcome Assessment

Each patient was assigned a single principal indication after physician review of the medical record. The principal indication was adjudicated from clinical notes, lesion distribution, treatment intent, and diagnostic information rather than prescription code alone. Initial chart abstraction was performed by one dermatologist (J.L.) and subsequently reviewed with the supervising dermatologist (J.P.); ambiguous cases were resolved by consensus discussion, and no case remained unresolved. This approach was used because administrative or billing diagnoses did not always reflect the primary reason for alitretinoin treatment.

Treatment response was assessed using chart-derived Investigator Global Assessment (IGA), with treatment success defined as final IGA 0 (clear) or 1 (almost clear). IGA was not prospectively recorded as a formal scale at each visit by the treating physicians; instead, both a baseline and a final IGA were retrospectively assigned by the reviewing dermatologists from the documented lesion description at the first alitretinoin prescription visit and at the last treatment-related visit, respectively, using predefined rules applied uniformly across indications. IGA 0 corresponded to no active or clinically relevant residual lesion documented, IGA 1 to minimal residual disease or near-complete clearance, and IGA 2 or higher to persistent active disease requiring further treatment or documented partial or absent response. Because adequate final documentation was a prerequisite for cohort entry, a final IGA could be assigned for all 99 patients. For CHE, PPP, and other inflammatory or keratinizing dermatoses, this measure was used as a pragmatic retrospective endpoint. For MF, formal CTCL response measures such as modified Severity-Weighted Assessment Tool, ISCL/EORTC response criteria, or time to next treatment were not consistently available; therefore, MF outcomes were summarized using the same conservative chart-derived IGA endpoint and analyzed only as exploratory findings. A uniform IGA 0/1 criterion permitted a single cross-indication summary but is not equivalent to disease-specific instruments such as PPPASI or ISCL/EORTC criteria, and it is expected to underestimate benefit in diseases for which partial response is clinically meaningful; response rates should therefore not be compared directly between indications.

Prescription persistence was defined as the interval between the first and last alitretinoin prescriptions. Actual treatment duration was recorded separately from chart review when clinical notes clarified treatment continuation, interruption, rechallenge, or meaningful exposure. Treatment response analyses used actual treatment duration, whereas persistence was used to describe prescribing behavior. Adverse events were counted when the Adverse Drug Reaction (ADR) Probability Scale indicated possible-or-greater attribution. Reasons for discontinuation and serial triglyceride and total cholesterol values were extracted from the medical record. Several measures were used to limit bias. The effective-treatment cohort was defined a priori rather than after outcomes were known; principal indication was adjudicated from clinical documentation rather than administrative or billing codes; adverse events were attributed using a formal causality scale rather than unstructured chart impression; and a single conservative dichotomous IGA endpoint was applied uniformly across all indications, including those for which disease-specific instruments would ordinarily be used.

### 2.4. Statistical Analysis and Ethics

Continuous variables are summarized as mean ± sample standard deviation and/or median, and categorical variables as counts and percentages. Initial alitretinoin dose was derived from the prescription-level extraction as the capsule strength dispensed on each patient’s first alitretinoin prescription date. Treatment success by initial alitretinoin dose was compared using Fisher exact test. Paired baseline and last available on-treatment lipid values were evaluated using the paired *t*-test, and the *p* values reported for mean lipid change correspond to this test; Wilcoxon signed-rank tests were performed as nonparametric sensitivity analyses. Lipid changes are reported as mean ± standard deviation with 95% confidence intervals for the mean change, and the proportions of patients exceeding conventional lipid thresholds were summarized descriptively. To address confounding in the unadjusted initial-dose comparison, an exploratory multivariable Firth penalized logistic regression was fitted with final IGA 0/1 as the outcome and initial dose, age, sex, and indication group (CHE versus off-label) as covariates; a secondary model additionally included actual treatment duration, which is a post-baseline variable and may act as a mediator rather than a confounder. Firth penalization was chosen because of the imbalanced dose groups and sparse strata. Odds ratios are reported with profile penalized-likelihood confidence intervals and penalized likelihood ratio *p* values. A two-sided *p* value < 0.05 was considered statistically significant. All analyses were performed using R version 4.6.1 (2026-06-24; R Foundation for Statistical Computing, Vienna, Austria). All data were de-identified. The study was approved by the Institutional Review Board of Daegu Catholic University Medical Center (approval number DCUMC IRB 2026-07-019; expedited review; approved on 15 July 2026) and was conducted in accordance with the Declaration of Helsinki. The requirement for informed consent was waived by the Institutional Review Board owing to the retrospective nature of the study and the use of de-identified data. IGA was dichotomized at 0/1 rather than modelled as an ordinal score because retrospective documentation supported a clear or almost-clear judgement more reliably than a full ordinal grade, and initial dose was grouped by the capsule strength dispensed on the first prescription date, no patient having received both strengths on that date. Subgroup analyses by indication and by initial dose were exploratory, were not adjusted for multiplicity, and were not powered for disease-specific inference. All analyses were complete-case without imputation; principal indication, IGA, actual treatment duration, and initial dose were available for all 99 patients, whereas paired lipid values were available for 88 patients for triglyceride and 90 for total cholesterol.

## 3. Results

### 3.1. Cohort and Indications

Over 10 years, 161 unique patients were prescribed oral alitretinoin; 38 (23.6%) received only a single prescription. Of the remaining 123 patients with more than one prescription date, 24 did not meet the predefined 1-month evaluable-course criterion owing to short exposure or insufficient follow-up documentation, leaving 99 patients in the effective-treatment cohort (50 women, 49 men; mean age 50.7 ± 16.0 years; range 16–89). Characteristics of the two excluded groups are shown in Supplementary Table S1. CHE was the leading indication (70/99, 70.7%), while off-label indications accounted for 29.3% of the cohort and included PPP, MF, parapsoriasis, porokeratosis, palmoplantar keratoderma, and Darier disease (Table 1). With respect to dose, 83 patients began treatment at 30 mg and 16 began at 10 mg; 44 patients received both the 10 mg and the 30 mg strength at some point during therapy. Initiation at 10 mg was more frequent among patients treated for off-label indications (10/29, 34.5%) than among patients with CHE (6/70, 8.6%).

### 3.2. Treatment Response

The overall treatment success rate (final IGA 0/1) was 66.7% (66/99) over a mean actual treatment duration of 12.4 months (Table 2). CHE showed the highest success rate (54/70, 77.1%). Responses in off-label indications were more variable: success occurred in 6 of 14 patients with PPP (42.9%) and 2 of 6 patients with MF (33.3%). Small subgroups such as parapsoriasis and porokeratosis showed responses in selected patients but were too small for disease-specific inference. Individual characteristics of all 29 off-label cases, including baseline and final IGA, initial dose, dose-exposure pattern, actual treatment duration, adverse events, and reason for discontinuation, are provided in Supplementary Table S2.

### 3.3. Dose and Response

Treatment response by initial dose is shown in Table 3. In the unadjusted comparison, patients who began at 30 mg were more likely to achieve final IGA 0/1 than those started at 10 mg (59/83, 71.1% vs. 7/16, 43.8%; Fisher exact test, *p* = 0.044), despite similar mean actual treatment durations. Among the 44 patients who received both strengths, 38 began at 30 mg and subsequently received 10 mg, whereas 6 began at 10 mg and subsequently received 30 mg (Supplementary Table S3). Success was highest in patients who started at 30 mg and later also received 10 mg (31/38, 81.6%), a pattern consistent with dose reduction following clinical improvement rather than with a benefit of switching. When the analysis was restricted to patients who received only their initial strength throughout treatment, the difference was attenuated and no longer significant (30 mg, 28/45, 62.2% vs. 10 mg, 5/10, 50.0%; Fisher exact test, *p* = 0.498). In exploratory Firth penalized logistic regression adjusting for age, sex, and indication group, initial dose was not independently associated with treatment success (odds ratio 1.84; 95% profile-likelihood confidence interval 0.56–5.90; *p* = 0.309), whereas off-label indication was associated with lower success (odds ratio 0.26; 95% confidence interval 0.10–0.66; *p* = 0.005); results were materially unchanged when actual treatment duration was added (Supplementary Table S5). These findings indicate that the unadjusted initial-dose association is substantially confounded by indication and by response-driven dose adjustment, and it should therefore be interpreted as exploratory rather than causal.

### 3.4. Safety and Lipid Changes

Adverse events of possible-or-greater probability were recorded in 21 patients (21.2%). The most common were dyslipidemia (*n* = 12), headache (*n* = 7), and gastrointestinal disturbance (*n* = 2); xerosis cutis (*n* = 2), arthralgia (*n* = 1), and erectile dysfunction (*n* = 1) were less frequent (Table 4). No unexpected safety signal was identified in chart review. Laboratory monitoring demonstrated statistically significant increases in both triglyceride and total cholesterol (Table 5). Mean triglyceride increased from 124.3 to 166.4 mg/dL (mean change +42.1 mg/dL; 95% CI, +24.8 to +59.5), and mean total cholesterol increased from 165.9 to 180.0 mg/dL (mean change +14.0 mg/dL; 95% CI, +7.0 to +21.1); both changes were significant by paired *t*-test (*p* < 0.001), with concordant Wilcoxon signed-rank results. At the last on-treatment measurement, triglyceride was ≥150, ≥200, ≥300, and ≥500 mg/dL in 38/88 (43.2%), 18/88 (20.5%), 9/88 (10.2%), and 3/88 (3.4%) patients, respectively, and total cholesterol was ≥200 and ≥240 mg/dL in 36/90 (40.0%) and 8/90 (8.9%) patients. Among patients with normal baseline values, new triglyceride elevation ≥150 mg/dL occurred in 16/62 (25.8%) and new total cholesterol elevation ≥200 mg/dL in 19/69 (27.5%) (Supplementary Table S4).

### 3.5. Treatment Discontinuation

Thirty patients (30.3%) discontinued alitretinoin (Table 6). Lack of effectiveness was the most frequent reason (*n* = 24), followed by dyslipidemia (*n* = 7) and headache (*n* = 3). Discontinuation because of abdominal pain, arthralgia, erectile dysfunction, xerosis, switch to tralokinumab for concomitant atopic dermatitis, or follow-up loss occurred in single cases. Reasons could overlap when more than one factor contributed to cessation.

## 4. Discussion

In this 10-year single-center Korean study, oral alitretinoin was used primarily for CHE but also for a heterogeneous group of selected off-label dermatoses. Among patients with an evaluable treatment course, two-thirds achieved final IGA 0/1. The strongest and most clinically interpretable result was observed in CHE, where 77.1% achieved clear or almost clear status. In contrast, outcomes in PPP, MF, and other off-label indications were variable and should be considered exploratory.

The favorable CHE outcome is consistent with prior evidence supporting alitretinoin in severe refractory CHE [3,4,5,6,7,8]. However, the 77.1% success rate in our cohort is higher than some large real-world studies, including PASSION and FUGETTA, which reported clear/almost clear rates closer to approximately one-third to one-half depending on analytic method and visit definition [5,6]. Several differences may explain this discrepancy. Our cohort excluded single-prescription and non-evaluable short-course patients, included longer real-world treatment exposure than 24-week observational studies, and relied on retrospective chart-derived IGA rather than protocolized prospective PGA assessment. Therefore, our CHE results should be interpreted as effectiveness among patients who continued sufficiently long to be evaluable, not as an intention-to-treat estimate for all patients who were prescribed alitretinoin.

PPP represented the most common off-label inflammatory indication. The PPP success rate of 42.9% resembles the more modest response reported in the Korean HE/PPP study, in which PPP responded less consistently than hyperkeratotic hand eczema [8]. This finding also fits the broader literature, where alitretinoin for PPP remains controversial. A randomized phase II trial found no significant advantage over placebo for PPPASI change, although interpretation has been complicated by a high placebo response and small sample size [9,10]. Taken together, our data do not establish alitretinoin as a general PPP treatment, but suggest that selected patients may benefit, particularly when lesions share hyperkeratotic morphology with alitretinoin-responsive CHE phenotypes.

The MF subgroup requires particular caution. Previous CTCL series have evaluated alitretinoin using CR/PR/SD/PD categories, disease stage, concomitant therapy, and sometimes time to next treatment [11,12,13]. In contrast, our retrospective dataset did not contain standardized CTCL response measures such as mSWAT or ISCL/EORTC response criteria. We therefore summarized MF using a conservative chart-derived IGA 0/1 endpoint, which may underrepresent partial response or stable disease and is not directly comparable with CTCL-specific studies. The observed MF success rate of 33.3% should therefore be viewed as descriptive evidence of selected clinical benefit rather than proof of CTCL efficacy.

The remaining off-label indications were small but not without precedent. Neither patient with palmoplantar keratoderma achieved IGA 0/1 despite published reports of benefit in refractory and punctate variants [16,17]; both courses were short (3 and 5 months), so insufficient exposure cannot be excluded. The single patient with Darier disease also did not respond over 36 months, a result more in keeping with the cautionary appraisal that followed early favorable case reports [20] than with the reports themselves [18]. Two of three patients with porokeratosis achieved clear or almost clear status, whereas the one patient with disseminated superficial actinic porokeratosis did not respond during a 4-month course, in contrast to an earlier Korean report of oral alitretinoin in this condition [19]. Parapsoriasis, which is generally managed within the MF spectrum, showed response in two of three patients and is therefore best interpreted alongside the CTCL subgroup [11,12,13]. With one to three patients per condition, these observations are descriptive only and cannot support disease-specific conclusions.

The unadjusted association between higher initial dose and better treatment success was directionally consistent with dose-response patterns reported in the CHE literature [3,4,7], but it did not withstand adjustment. Patients treated for off-label indications, who had substantially lower success rates, were four times more likely to be started at 10 mg than patients with CHE, and among patients who received both strengths the highest success rate occurred in those who started at 30 mg and were later also given 10 mg—a sequence more consistent with dose reduction after clinical improvement than with any benefit of the lower dose. Accordingly, in a restricted analysis of patients maintained on their initial strength and in Firth penalized logistic regression adjusting for age, sex, and indication, initial dose was no longer significantly associated with response. We therefore do not interpret our data as evidence of an independent dose-response effect. Dose was not randomized, and physicians may have selected 10 mg for older, frailer, lipid-prone, or otherwise higher-risk patients, or for indications considered exploratory. Initial dose should be regarded as a marker of prescribing context rather than an independent determinant of outcome, and prospective evaluation with protocolized dosing would be required to establish a true dose-response relationship.

The safety profile was manageable and dominated by lipid abnormalities and headache, consistent with known retinoid effects and previous alitretinoin studies [3,5,6,8]. A clinically recorded dyslipidemia event occurred in 12 patients, but paired laboratory analysis revealed broader average increases in triglyceride and total cholesterol. This distinction is important: many patients may experience measurable lipid elevation without it being coded as an adverse event or leading to discontinuation. Routine lipid monitoring remains important, especially for long-term treatment and off-label use where treatment duration may extend beyond standard CHE courses.

Lack of effectiveness, rather than toxicity, was the leading reason for discontinuation. This pattern suggests that for patients who tolerate alitretinoin, the major limitation in real-world practice is incomplete or absent response. It also reinforces the need to define treatment goals and stopping rules, particularly for off-label indications with limited evidence.

This study has several limitations. It was retrospective and single-center, and several indication subgroups were small. There was no untreated or comparator group. Principal indication and IGA were assigned by chart review and therefore subject to documentation bias. Disease-specific outcome instruments, including PPPASI for PPP and mSWAT/ISCL response criteria for MF, were not consistently available. Concomitant topical, systemic, and phototherapeutic treatments were not systematically recorded in the study dataset, and prior treatment history, lesion morphology, smoking status in PPP, and relapse after discontinuation were likewise incompletely captured; the observed responses therefore cannot be attributed to alitretinoin alone, and this limitation is most consequential for the off-label subgroups, particularly MF, in which skin-directed and systemic co-treatments are routine. A formal time-to-response analysis was not performed because visit intervals were not standardized over the 10-year period and the first visit at which IGA 0/1 was reached was not consistently documented; reported outcomes therefore reflect status at the end of the available treatment course rather than the speed of response. Finally, the effective-treatment cohort design improves interpretability of treatment-course outcomes but may overestimate response compared with an intention-to-treat prescribing population. Because this was a single-center Korean tertiary-care cohort, the findings may be most applicable to similar dermatology practice settings and may not be generalizable to community clinics or non-Korean populations.

Despite these limitations, this study provides a decade-long overview of how alitretinoin was used in routine Korean dermatology practice. The data support its real-world effectiveness in CHE, confirm the need for lipid monitoring, and provide cautious descriptive evidence that selected off-label patients may benefit. Future prospective studies should incorporate disease-specific severity measures, standardized response definitions, morphology-based subgrouping, and systematic safety monitoring.

## 5. Conclusions

In this 10-year real-world Korean cohort, oral alitretinoin showed favorable effectiveness in chronic hand eczema and variable exploratory responses in selected off-label dermatoses. Initiation at 30 mg was associated with higher treatment success than initiation at 10 mg in unadjusted analysis, but this association did not persist after restriction to patients maintained on their initial dose or after multivariable adjustment, and it appears to be confounded by indication and by response-driven dose adjustment. Lipid elevation was common on paired laboratory monitoring, with new triglyceride or total cholesterol elevation above conventional thresholds in approximately one-quarter of patients with normal baseline values, underscoring the importance of routine surveillance. Off-label use in PPP, MF, and other dermatoses should be considered hypothesis-generating rather than definitive.

## Figures and Tables

**Table 1:** Indications, demographics, and prescription persistence in the effective-treatment cohort (*n* = 99).

Principal Indication	*n*	F/M	Age, Mean ± SD (Range)	Prescription Persistence, Median (mo)
Chronic hand eczema	70	36/34	49.8 ± 14.8 (19–80)	6.5
Palmoplantar pustulosis	14	9/5	54.3 ± 17.9 (16–89)	8.1
Mycosis fungoides	6	2/4	46.7 ± 21.3 (21–84)	22.5
Parapsoriasis	3	0/3	59.0 ± 12.5 (47–72)	14.7
Porokeratosis *	3	1/2	69.3 ± 10.6 (58–79)	14.5
Palmoplantar keratoderma	2	1/1	39.0 ± 24.0 (22–56)	31.9
Darier disease	1	1/0	28	31.6
Total	99	50/49	50.7 ± 16.0 (16–89)	6.8

* Includes one patient with disseminated superficial actinic porokeratosis. Prescription persistence was defined as the interval between first and last prescription and should be distinguished from actual treatment duration used for response analysis.

**Table 2:** Treatment success and actual treatment duration by indication.

Principal Indication	*n*	Success, *n* (%)	Mean Actual Treatment Duration (mo)
Chronic hand eczema	70	54 (77.1)	11.7
Parapsoriasis	3	2 (66.7)	31.7
Porokeratosis	3	2 (66.7)	12.0
Palmoplantar pustulosis	14	6 (42.9)	9.1
Mycosis fungoides	6	2 (33.3)	17.3
Palmoplantar keratoderma	2	0 (0)	4.0
Darier disease	1	0 (0)	36.0
Total	99	66 (66.7)	12.4

**Table 3:** Treatment success by initial alitretinoin dose.

Initial Dose	*n*	Success, *n* (%)	Mean Actual Treatment Duration (mo)
30 mg	83	59 (71.1)	12.2
10 mg	16	7 (43.8)	13.2
Total	99	66 (66.7)	12.4

Initial dose was defined as the strength of the alitretinoin prescription issued on the date of the first alitretinoin prescription, determined from the prescription-level extraction; no patient received both strengths on that date. 30 mg versus 10 mg success: Fisher exact test, *p* = 0.044.

**Table 4:** Adverse events attributed as ADR scale possible or greater.

Adverse Event	*n* (F/M)	Led to Discontinuation
Dyslipidemia	12 (4/8)	7
Headache	7 (2/5)	3
Gastrointestinal disturbance	2 (1/1)	1
Xerosis cutis	2 (1/1)	1
Arthralgia	1 (0/1)	1
Erectile dysfunction	1 (0/1)	1
Any adverse event	21 (6/15)	12
None recorded	78 (44/34)	-

Categories are not mutually exclusive; one patient could have more than one adverse event or discontinuation reason.

**Table 5:** Lipid changes during treatment among patients with paired values.

Parameter (mg/dL)	*n*	Baseline, Mean ± SD	Last On-Treatment, Mean ± SD	Mean Change (95% CI)	Paired *t*-Test *p* Value	Wilcoxon *p* Value
Triglyceride	88	124.3 ± 58.5	166.4 ± 109.4	+42.1 (+24.8 to +59.5)	<0.001	<0.001
Total cholesterol	90	165.9 ± 43.9	180.0 ± 46.7	+14.0 (+7.0 to +21.1)	<0.001	<0.001

Patients without paired lipid testing were excluded from this analysis; denominators reflect available paired values. The *p* values shown for mean change correspond to paired *t*-tests; Wilcoxon signed-rank tests were performed as nonparametric sensitivity analyses and were concordant. CI, confidence interval; SD, standard deviation.

**Table 6:** Reasons for treatment discontinuation (*n* = 30; reasons may overlap).

Reason	No. of Patients
Lack of effectiveness	24
Dyslipidemia	7
Headache	3
Abdominal pain	1
Arthralgia	1
Erectile dysfunction	1
Xerosis cutis	1
Switched to tralokinumab (atopic dermatitis)	1
Follow-up loss	1

## Data Availability

The data presented in this study are available on reasonable request from the corresponding author. The data are not publicly available because of privacy and ethical restrictions on patient records.

## Supplementary Materials

The following supporting information can be downloaded at: https://www.mdpi.com/article/10.3390/medicina62091625/s1, Table S1: Characteristics of the excluded groups and the effective-treatment cohort; Table S2: Individual off-label cases in the effective-treatment cohort (*n* = 29).; Table S3: Initial dose and dose-exposure patterns; Table S4: Clinically relevant lipid thresholds during treatment; Table S5: Exploratory Firth penalized logistic regression for treatment success (final IGA 0/1).
