# Supplementary material for: Real-World Effectiveness and Safety of Oral Alitretinoin in Chronic Hand Eczema with Exploratory Off-Label Indications: A 10-Year Single-Center Korean Study
Source: Medicina (Kaunas). 2026 Aug 24;62(9):1625. doi: 10.3390/medicina62091625 (PMC13609635; doi:10.3390/medicina62091625)
Supplement: Supplementary file 1 [file medicina-62-01625-s001.zip › medicina-4499184-supplementary.pdf]

Supplementary Materials

Real-World Effectiveness and Safety of Oral Alitretinoin in Chronic Hand Eczema with Exploratory Off-Label Indications: A 10-Year Single-Center Korean Study

Table S1. Characteristics of the excluded groups and the effective-treatment cohort.

Group	n	F / M	Age, mean ± SD (range)	Median no. of prescription dates	Median prescription span (mo)	Analysis status
Single prescription date	38	19 / 19	45.8 ± 14.8 (18–73)	1	0.0	Excluded from outcome analysis
≥ 2 prescription dates but non-evaluable (< 1-month course)	24	14 / 10	50.0 ± 14.0 (19–70)	2	0.4	Excluded from outcome analysis
Effective-treatment cohort	99	50 / 49	50.7 ± 16.0 (16–89)	9	6.8	Included in outcome analysis

Prescription span was defined as the interval between the first and last prescription date. Reasons for non-continuation after a single prescription were not consistently documented in the medical record and were therefore not classified; response and adverse-event status were not inferred from a single prescription alone.

Table S2. Individual off-label cases in the effective-treatment cohort (n = 29).

Case	Diagnosis	Sex/Age	Baseline IGA	Final IGA	Success	Initial dose	Dose exposure	Duration (mo)	Adverse event(s)	Discontinuation
1	Palmoplantar pustulosis	F/16	2	1	Yes	30 mg	30 mg only	10	None	Continued
2	Palmoplantar pustulosis	M/30	2	1	Yes	30 mg	Both strengths	25	None	Continued
3	Palmoplantar pustulosis	F/36	2	2	No	30 mg	30 mg only	4	None	Discontinued: lack of effectiveness
4	Palmoplantar pustulosis	M/50	3	2	No	30 mg	30 mg only	6	None	Discontinued: lack of effectiveness
5	Palmoplantar pustulosis	F/52	3	2	No	30 mg	Both strengths	7	None	Discontinued: lack of effectiveness
6	Palmoplantar pustulosis	F/53	2	2	No	30 mg	30 mg only	12	None	Continued
7	Palmoplantar pustulosis	M/55	2	1	Yes	30 mg	30 mg only	6	None	Continued
8	Palmoplantar pustulosis	F/58	3	2	No	30 mg	Both strengths	4	None	Continued
9	Palmoplantar pustulosis	M/58	3	2	No	10 mg	10 mg only	3	dyslipidemia	Discontinued: lack of effectiveness, dyslipidemia
10	Palmoplantar pustulosis	F/64	2	2	No	10 mg	10 mg only	2	dyslipidemia, headache	Discontinued: lack of effectiveness, dyslipidemia, headache

Case	Diagnosis	Sex/Age	Baseline IGA	Final IGA	Success	Initial dose	Dose exposure	Duration (mo)	Adverse event(s)	Discontinuation
11	Palmoplantar pustulosis	F/65	3	1	Yes	30 mg	30 mg only	24	None	Continued
12	Palmoplantar pustulosis	F/67	3	1	Yes	30 mg	30 mg only	8	None	Continued
13	Palmoplantar pustulosis	M/67	2	0	Yes	10 mg	10 mg only	4	None	Continued
14	Palmoplantar pustulosis	F/89	3	2	No	10 mg	10 mg only	13	None	Continued
15	Mycosis fungoides	M/21	3	2	No	30 mg	30 mg only	6	erectile dysfunction	Discontinued: lack of effectiveness, erectile dysfunction
16	Mycosis fungoides	F/33	2	1	Yes	30 mg	30 mg only	9	None	Continued
17	Mycosis fungoides	M/43	3	2	No	30 mg	Both strengths	24	None	Continued
18	Mycosis fungoides	M/48	3	2	No	10 mg	Both strengths	14	None	Discontinued: lack of effectiveness
19	Mycosis fungoides	F/51	3	1	Yes	30 mg	Both strengths	48	None	Continued
20	Mycosis fungoides	M/84	4	3	No	30 mg	30 mg only	3	None	Discontinued: lack of effectiveness
21	Parapsoriasis	M/47	3	1	Yes	30 mg	30 mg only	16	dyslipidemia	Continued
22	Parapsoriasis	M/58	3	2	No	30 mg	Both strengths	7	dyslipidemia, headache	Continued
23	Parapsoriasis	M/72	3	1	Yes	30 mg	Both strengths	72	None	Continued
24	Porokeratosis	M/58	3	1	Yes	10 mg	10 mg only	24	None	Continued
25	Porokeratosis	F/71	3	1	Yes	10 mg	10 mg only	8	dyslipidemia	Continued
26	Porokeratosis (DSAP)	M/79	3	3	No	10 mg	Both strengths	4	None	Discontinued: lack of effectiveness
27	Palmoplantar keratoderma	M/22	3	3	No	10 mg	10 mg only	5	None	Discontinued: lack of effectiveness
28	Palmoplantar keratoderma	F/56	2	2	No	10 mg	10 mg only	3	dyslipidemia	Discontinued: lack of effectiveness, dyslipidemia
29	Darier disease	F/28	4	3	No	30 mg	30 mg only	36	None	Discontinued: lack of effectiveness

IGA, Investigator Global Assessment; treatment success was defined as final IGA 0 or 1. Dose exposure indicates which capsule strengths were dispensed at any time during treatment; sequence within the "both strengths" category was inferred from the strength dispensed on the first prescription date. Concomitant topical, systemic, and phototherapeutic treatments were not systematically recorded in the study dataset and are therefore not reported; responses cannot be attributed to alitretinoin alone.

Table S3. Initial dose and dose-exposure patterns.

Dose exposure pattern	Initial dose	n	Success, n (%)	Mean actual duration (mo)
30 mg strength only	30 mg	45	28 (62.2)	7.6
10 mg strength only	10 mg	10	5 (50.0)	7.3
Both strengths	30 mg	38	31 (81.6)	17.8
Both strengths	10 mg	6	2 (33.3)	23.2
Total	—	99	66 (66.7)	12.4

Initial dose was defined as the capsule strength dispensed on the first alitretinoin prescription date; no patient received both strengths on that date. This table describes exposure patterns only. The high success rate among patients who started at 30 mg and later also received 10 mg is consistent with dose reduction following clinical improvement, and this table should therefore not be read as an adjusted dose-response analysis.

Table S4. Clinically relevant lipid thresholds during treatment.

Parameter	Threshold	n	Denominator	%
Triglyceride	Last on-treatment ≥ 150 mg/dL	38	88	43.2
	Last on-treatment ≥ 200 mg/dL	18	88	20.5
	Last on-treatment ≥ 300 mg/dL	9	88	10.2
	Last on-treatment ≥ 500 mg/dL	3	88	3.4
	New elevation to ≥ 150 mg/dL (baseline < 150 mg/dL)	16	62	25.8
Total cholesterol	Last on-treatment ≥ 200 mg/dL	36	90	40.0
	Last on-treatment ≥ 240 mg/dL	8	90	8.9
	New elevation to ≥ 200 mg/dL (baseline < 200 mg/dL)	19	69	27.5

Threshold analyses were restricted to patients with paired lipid values (triglyceride n = 88; total cholesterol n = 90). Conventional reference thresholds were triglyceride ≥ 150 mg/dL and total cholesterol ≥ 200 mg/dL; higher thresholds are shown to indicate greater clinical relevance.

Table S5. Exploratory Firth penalized logistic regression for treatment success (final IGA 0/1).

Model A — baseline covariates only (n = 99; 66 events)

Covariate	Odds ratio	95% profile-likelihood CI	p value
Initial dose 30 mg (vs 10 mg)	1.84	0.56 – 5.90	0.309
Age (per year)	1.00	0.97 – 1.03	0.917
Male sex (vs female)	0.64	0.26 – 1.53	0.311
Off-label indication (vs CHE)	0.26	0.10 – 0.66	0.005

Model B — additionally adjusted for actual treatment duration

Covariate	Odds ratio	95% profile-likelihood CI	p value
Initial dose 30 mg (vs 10 mg)	1.53	0.42 – 5.47	0.511
Age (per year)	1.00	0.97 – 1.02	0.778
Male sex (vs female)	0.64	0.25 – 1.58	0.330
Off-label indication (vs CHE)	0.20	0.07 – 0.56	0.002
Actual treatment duration (per month)	1.06	1.01 – 1.13	0.017

Firth penalization was used because of the imbalanced initial-dose groups and sparse strata. Odds ratios are presented with profile penalized-likelihood confidence intervals and penalized likelihood ratio p values. Actual treatment duration is a post-baseline variable that may act as a mediator rather than a confounder and is therefore presented only as a secondary model. CHE, chronic hand eczema; CI, confidence interval.
